# Metabolic constraints for a novel symbiosis

**DOI:** 10.1098/rsos.150708

**Published:** 2016-03-23

**Authors:** Megan E. S. Sørensen, Duncan D. Cameron, Michael A. Brockhurst, A. Jamie Wood

**Affiliations:** 1 Department of Biology, University of York, York YO10 5GG, UK; 2 Department of Mathematics, University of York, York YO10 5GG, UK; 3 Department of Animal and Plant Sciences, University of Sheffield, Western Bank, Sheffield S10 2TN, UK

**Keywords:** metabolism, endosymbiosis, cyanobacteria

## Abstract

Ancient evolutionary events are difficult to study because their current products are derived forms altered by millions of years of adaptation. The primary endosymbiotic event formed the first photosynthetic eukaryote resulting in both plants and algae, with vast consequences for life on Earth. The evolutionary time that passed since this event means the dominant mechanisms and changes that were required are obscured. Synthetic symbioses such as the novel interaction between *Paramecium bursaria* and the cyanobacterium *Synechocystis* PC6803, recently established in the laboratory, permit a unique window on the possible early trajectories of this critical evolutionary event. Here, we apply metabolic modelling, using flux balance analysis (FBA), to predict the metabolic adaptations necessary for this previously free-living symbiont to transition to the endosymbiotic niche. By enforcing reciprocal nutrient trading, we are able to predict the most efficient exchange nutrients for both host and symbiont. During the transition from free-living to obligate symbiosis, it is likely that the trading parameters will change over time, which leads in our model to discontinuous changes in the preferred exchange nutrients. Our results show the applicability of FBA modelling to ancient evolutionary transitions driven by metabolic exchanges, and predict how newly established endosymbioses, governed by conflict, will differ from a well-developed one that has reached a mutual-benefit state.

## 1. Introduction

Endosymbiosis, a symbiotic relationship where one organism resides within another, has led to some of the most important transitions in the evolution of eukaryotes, including their origin and later the formation of photosynthetic eukaryotes [1]. The endosymbiotic origin of organelles, conceived by Merechowsky [2], was a controversial concept, but championed by Margulis [3] it was eventually accepted with the advent of molecular techniques [4–7]. Endosymbiosis is a common occurrence in algae, though the evolutionary transition of photosynthetic symbionts into organelles is rare [8]. The primary endosymbiosis event underpinning the evolution of photosynthetic eukaryotes involved the acquisition of a cyanobacterium; this has since radiated, leading to the evolution of today's land plants and algae. Over the intervening hundreds of millions of years, the symbiont has experienced coevolution and genome reduction (the loss of genes required for free living) to the extent that it has lost autonomy and become an organelle—the chloroplast [9]. Moreover, genome reduction over the course of coevolution between host and endosymbiont is frequently accompanied by gene transfer from plastid to host nucleus, as has occurred in plants with the small subunit of the primary enzyme of carbon fixation ribulose-1,5-bisphosphate carboxylase/oxygenase (rbcS) encoded by the nuclear rather than the plastid genome with the large subunit, rbcL, remaining encoded by the plastid [10–12]. This highly derived form makes the establishment of this major endosymbiotic event difficult to study.

Recently, an artificial endosymbiosis was created by supplying aposymbiotic *Paramecium bursaria* with *Synechocystis* PCC6803 [13]. These organisms do not naturally form a symbiosis and so have not coevolved. *Synechocystis* is a cyanobacterium that requires nitrogen and fixes carbon and therefore is capable of acting as a photosymbiont for the ciliate. This discovery permits the recapitulation of the early evolution of the original endosymbiotic event. Synthetic symbioses of this kind potentially allow us to interrogate the evolutionary likelihood and possible initial trajectories of transitions to endosymbiosis. In the case of the partnership created by Ohkawa *et al.*, this is possible to an unusual level of detail, because the genome of the introduced symbiont is available to study and can be used to model the metabolism of the ancestral state.

*Paramecium bursaria* has an established endosymbiosis with the green algae *Chlorella* spp. This symbiosis sits on the borderline between facultative and obligate and is thus suggestive of the potential to establish novel endosymbioses. The *Chlorella* symbiont is vertically inherited and the two cell cycles are synchronized, which indicates a tightly coevolved relationship. However, in most natural isolates, both organisms can survive if separated, suggesting that this remains a facultative association. *Paramecium* and *Chlorella* have a classical photosymbiotic exchange, whereby the *Chlorella* provides organic carbon fixed by photosynthesis and *Paramecium* in return supplies organic nitrogen. It is estimated that the *Chlorella* endosymbionts release 57% of their fixed carbon to their host [14]. This partnership has been studied and thoroughly documented owing to the ease of isolation and reinfection [15]. The two organisms are interdependent to the extent that their circadian cycles are linked. For instance, it has been demonstrated that *P. bursaria*/*Chlorella* holobionts (the collective term for the endosymbiotic state) have a longer period than aposymbiont *P. bursaria* individuals, *P. bursaria* mutants with an arrhythmic circadian rhythm can be rescued by symbionts, and, if the host and symbiont have out of phase circadian rhythms, then *P. bursaria* will gradually shift its rhythm to match that of *Chlorella* [16].

It has been demonstrated that the disaccharide maltose constitutes the primary carbon exchange metabolite [17] from symbiont to host. It is provided during both day and night but by two different pathways: in the light, maltose is synthesized de novo from the products of the Calvin cycle, whereas in the dark, it is generated from starch degradation via the enzyme amylase [17]. In coevolved partnerships such as the *P. bursaria*/*Chlorella* holobiont, the exchange is not a passive process as evidenced by inhibition of serine uptake into *Chlorella* by host Ca^2+^ coupled to the observation that host glucose increases the uptake of serine by *Chlorella* [18,19]. While the basis of carbon metabolism and transport to the holobiont is well resolved, the mechanistic basis for the reciprocal transfer of nitrogen to the endosymbiont is not yet verified, though there are several potential processes. Amino acids have been suggested as candidate nitrogen transfer molecules as the Japanese *Chlorella* strain F36-ZK that has lost its nitrate reductase activity remains able to use amino acids [19]. Alternatively, other work suggests that *Paramecium* produces nitrogenous waste in the form of nucleic acid derivatives, such as guanine and xanthine [20], which are then assimilated by *Chlorella* [21]. Nucleoside recycling has been demonstrated in other endosymbioses [22,23], and the efficiency of using a host waste product would decrease the cost of symbiosis.

The interchange of metabolites between host and symbiont is key to understanding the evolutionary mechanisms for symbiosis formation. The metabolic exchange between the ciliate and *Synechocystis* in the novel interaction reported by Ohkawa *et al.* [13] is unlikely to be identical to that between *P. bursaria* and *Chlorella*, because the maltose exporter is an Archaeplastida innovation and there is no evidence to suggest *Synechocystis* can produce maltose [24,25]. The exchanges, however, are probably similar, because *Paramecium*'s recognition of potential symbionts will most likely require a supply of certain metabolites.

To capture the metabolic potential of the symbiotic partners, we require a detailed model capable of capturing the metabolic exchanges and changes in the evolution. A powerful theoretical method for analysing metabolism is flux balance analysis (FBA), which is capable of predicting the optimal metabolic fluxes of an organism and thus its growth rate [26–28]. Within the constraints of stoichiometry, FBA calculates the flux through each known reaction in the cell. The flux values are optimized with respect to the objective function. This varies, but is commonly taken as the organism's biomass on the assumption that organisms ‘prioritize’ growth and division. The model requires a large amount of data and so is limited to organisms with in-depth metabolic and genomic information. Furthermore, the enzymes and genes are considered to be Boolean values (they are ‘on’ or ‘off’); therefore, there is no regulation, and it assumes no underlying constraints preventing optimality. Despite its simplifying assumptions, FBA has significant applications in biotechnology [29] and in several cases has successfully predicted the outcome of evolution experiments [23,30,31]. Owing to its potential for biotechnology, several FBA models have been created for *Synechocystis* PCC6803, which is a very well characterized organism [32–34]. Unfortunately, there is insufficient data to create a complementary *Paramecium* FBA model, because its genetic complexity has prevented any whole genome sequencing.

To understand the establishment of endosymbiosis and therefore its evolution, evidence of the initial metabolic exchange between the host and symbiont is necessary. In this article, we use FBA modelling to predict the emergent metabolic trading in the synthetic endosymbiosis between *Synechocystis* PCC6803 and *Paramecium bursaria*.

## 2. Methods

We adopt the most recent FBA model of *Synechocystis* published by Knoop *et al.* [34] as our starting point. The model was then modified for a symbiosis by introducing an exchange reaction that forces nutrient exchange as detailed below. Arguably, endosymbionts satisfy more of the assumptions of FBA modelling than other organisms, because the host provides a stable environment for the symbiont permitting a context with less fluctuation in gene expression. Furthermore, obligate endosymbionts that have co-evolved with their host experience gene reduction and a decrease in transcriptional regulation, both of which makes FBA modelling more appropriate [23].

The FBA model used is the iHK677 model [34] augmented by the explicit inclusion of transport reactions. The iHK677 model encompasses 677 genes that encode for 759 reactions. The network defines six cellular compartments—the cytosol, plasma membrane, thylakoid membrane, thylakoid lumen, carboxysomes and periplasm—in addition to the extracellular space. The symbiotic exchange reaction was included when appropriate. Biomass was used as the objective function. A second optimization was applied that minimizes the reaction fluxes while maintaining the optimum biomass to remove futile cycles. The metabolic modelling was performed in a custom Java environment using the GLPK library for the linear optimization.

The only constraints on reaction fluxes were taken from Knoop *et al.* [34] and are: general adenosine triphosphate (ATP) consumption for cellular maintenance (0.13 mmol gDW^−1 ^h^−1^), a residual respiration rate (0.2263 mmol gDW^−1 ^h^−1^), Mehler-like reaction (0.2263 mmol gDW^−1 ^h^−1^), reactive oxygen species production at PSII (0.0477 mmol gDW^−1 ^h^−1^) and Mehler reaction at PSI (0.0473 mmol gDW^−1 ^h^−1^). In the standard condition, light is assumed to be the limiting factor and is set to 18.7 mmol gDW^−1 ^h^−1^ and nutrients are considered unlimited, though carbon uptake is restricted to bicarbonate (HCO_3_) and nitrogen uptake is as nitrate $\left({\mathrm{NO}}_{3}^{-}\right)$. The model includes the reactions for other sources but these have a default value of ‘off’.

When investigating different nitrogen sources a maximum uptake rate per nitrogen molecule was introduced to the model. A maximum uptake rate of 0.46 g N gDW^−1 ^d^−1^ was used that has been measured by Kim [35].

## 3. Results

**Figure 1.**
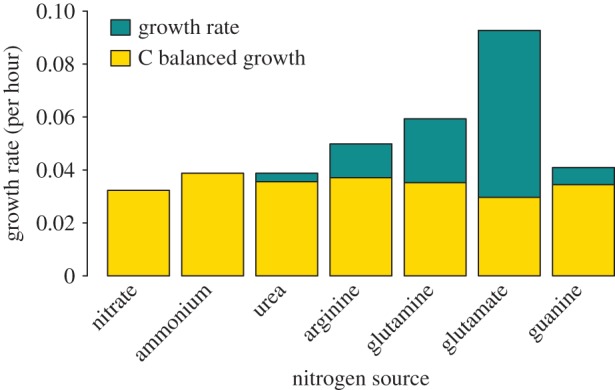
FBA predicted growth rates on different nitrogen sources in the standard condition and when carbon compensated.

Our first objective is to examine the potential of the *Synechocystis* model to uptake different nitrogen sources—the main exchange element received by this organism. Some of the nitrogen sources contain carbon and therefore the host, which is providing the nitrogen, is giving some carbon away in order to receive carbon. The initial model is for a free-living and therefore ‘selfish’ *Synechocystis*, which prefers the source that maximizes its growth. In this case, glutamate is strongly predicted as the best source for growth (figure 1). However, carbon compensation can be introduced to model a more mutualistic situation, in which the *Synechocystis* does not benefit from the carbon within the nitrogen source. When carbon compensation is applied (figure 1), the predicted growth rate across the nitrogen sources is similar, and the advantage of the amino acids, particularly glutamate, is no longer prominent compared with the free-living model. This is because the *Synechocystis* is no longer gaining the benefit of any carbon within the nitrogen source and glutamate has the highest C : N ratio. Under carbon compensation, arginine and ammonium act as the best nitrogen sources.

A symbiotic state was then created by including a complete exchange reaction: in order for *Synechocystis* to uptake nitrogen, it must export carbon. Two examples of which are shown below (reactions 770 and 772). A key parameter in defining these reactions is the ratio of carbon to nitrogen exchange, effectively the relative worth of these elements. This is a variable parameter which is determined both by the environmental context and by which partner is exerting control, i.e. determining the price for the exchange. In the examples below, we assume the host is in control as this is representing the initial establishment of the symbiosis. The value is therefore estimated using a C : N ratio from a related ciliate, *Paramecium caudatum*, which has a C : N ratio of 3.5 according to measurements by Finlay [36]. All calculations are based on the number of carbon or nitrogen molecules within the compound. For example, reaction 770 below shows the exchange requirement for the six-carbon glucose (3.5/6.0) in order for the single N-containing nitrate to be taken up and reaction 772 shows the exchange between the six-carbon glucose and the two-nitrogen one-carbon urea (((2*3.5) + 1)/6):

$$\begin{array}{rl} & \mathrm{Reaction}\;770:0.583*{\mathrm{glucose}}_{\left[\mathrm{cyt}\right]}+{\mathrm{nitrate}}_{\left[\mathrm{ext}\right]}+{\mathrm{ATP}}_{\left[\mathrm{cyt}\right]}+H_{2}O_{\left[\mathrm{cyt}\right]}\to \\ & \;0.583*{\mathrm{glucose}}_{\left[\mathrm{ext}\right]}+{\mathrm{nitrate}}_{\left[\mathrm{cyt}\right]}+{\mathrm{ADP}}_{\left[\mathrm{cyt}\right]}+{\mathrm{orthophosphate}}_{\left[\mathrm{cyt}\right]}\\ & \mathrm{Reaction}\;772:1.333*\;{\mathrm{glucose}}_{\left[\mathrm{cyt}\right]}+{\mathrm{urea}}_{\left[\mathrm{ext}\right]}+{\mathrm{ATP}}_{\left[\mathrm{cyt}\right]}+H_{2}O_{\left[\mathrm{cyt}\right]}\to \\ & \;1.333*{\mathrm{glucose}}_{\left[\mathrm{ext}\right]}+{\mathrm{urea}}_{\left[\mathrm{cyt}\right]}+{\mathrm{ADP}}_{\left[\mathrm{cyt}\right]}+{\mathrm{orthophosphate}}_{\left[\mathrm{cyt}\right]} \end{array}$$

**Figure 2.**
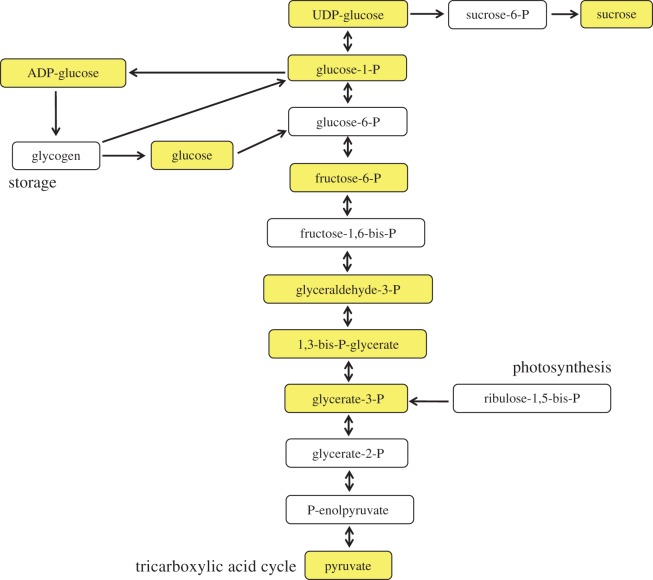
The chosen carbon compounds (yellow) in the context of metabolism.

The model was then used to predict the identity of the carbon export compound. Representative carbon compounds were chosen (figure 2) that span from the output of photosynthesis to the storage compound of *Synechocystis*, glycogen [37]. Pyruvate was also included because of its pivotal role in carbohydrate metabolism.

**Figure 3.**
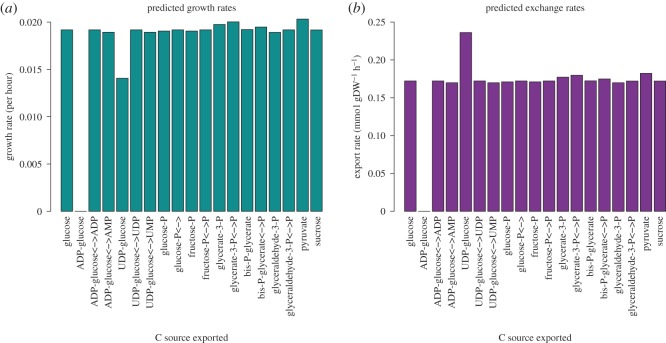
FBA predictions for different carbon export compounds. (*a*) The predicted growth rate values and (*b*) the predicted nitrogen uptake flux.

The selected compounds were first exchanged for the standard nitrogen source, nitrate. The predicted growth rates in this case have only small variation (figure 3*a*), but some salient features are apparent. For this analysis, any carbon compound containing phosphate was also tested in a phosphate antiporter situation. This allows for any phosphate to be regained, which otherwise increases the cost of the exchange. This is a plausible addition, because an antiport mechanism is theorized to have facilitated exchange in the primary endosymbiotic event [38], and phosphate antiporters are currently present in the exchange between chloroplast and the cytoplasm [39]. It is evident that the phosphate antiport makes a significant difference, especially for adenosine diphosphate (ADP)-glucose that cannot grow without it. The different uptake rates (figure 3*b*) suggest that the higher uptake is used as compensation for when there is no antiport mechanism. This is shown by uridine diphosphate (UDP)-glucose. Overall, pyruvate export leads to the highest growth rate of *Synechocystis* though the variation is small.

**Figure 4.**
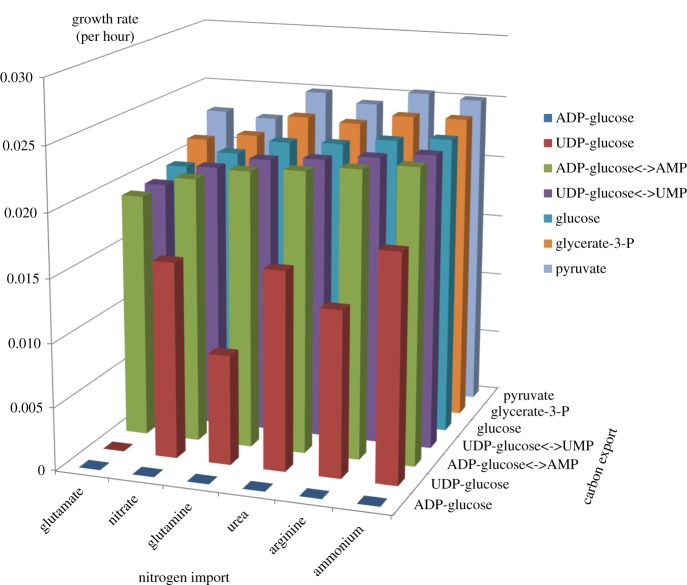
Predicted growth rates of the different combinations of the nitrogen import compounds and the carbon export compounds. The nitrogen sources have been arranged on the horizontal axis in order of increasing growth rate from left to right when exchanged for glucose, to allow easier comparisons.

The analysis was then expanded to consider the range of carbon sources in combination with the range of nitrogen sources (figure 4) for the symbiotic state. The results are not merely additive and the sum of the two previous tests, but rather some of the combinations also behave non-additively. Pyruvate is unusual because it is the only carbon source for which arginine and not ammonium results in the highest growth and for which glutamate does not lead to the lowest growth rate. UDP-glucose has much larger differences between the nitrogen sources, and there is no growth if it is exchanged for glutamate. This combinatorial analysis predicts that a pyruvate for arginine is the optimal exchange when the relationship is mutualistic.

This analysis was performed with a set C : N ratio that assumed the host was in control and therefore sets the relative value of the nutrients. This is the likely ‘endpoint’ in the endosymbiosis as the host could egest/digest any uncooperative symbionts that did not adhere to the ‘set price’. However, it may be that in the transitional stages the symbiont retains a degree of autonomy and therefore has more influence on the price.

**Figure 5.**
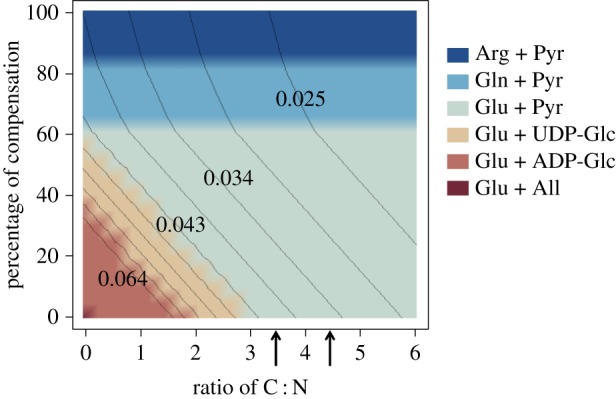
Optimal metabolite exchange across a range of ratios and degrees of carbon compensation (from 0 to 100%). The gradient lines indicate the value of *Synechocystis*'s growth rate. The space between a pair of contour lines represents a change in growth rate of 0.0045 mmol biomass gDW^−1^ h^−1^ and the growth rate is highest in the bottom left corner and lowest in the top right. The arrows indicate the C : N ratio of the two organisms; 3.5 is the value for the host and 4.5 for the symbiont.

To investigate the effect of the C : N ratio and therefore the price, the optimum metabolite exchange was identified over a range of ratios and also over a degree of carbon compensation (figure 5). As both the C : N ratio and carbon compensation increases, the symbiosis becomes more costly for the symbiont and more beneficial to the host. Interestingly, the transitions between the carbon sources are dependent on the C : N ratio, but the transitions between the nitrogen sources are not and instead occur at set percentages of compensation. As the ratio, and therefore the price, increases the transition to using pyruvate as the carbon export compound occurs more readily. Pyruvate contains no phosphate or nitrogen, unlike UDP and ADP; therefore, it may be that the cost of these additional molecules intensifies at higher ratio values.

The C : N ratio of *Synechocystis,* predicted in the biomass function of the metabolic model, is 4.5, whereas the host ratio used earlier is 3.5. The graph demonstrates that the change between these two values is very little, with no difference between their transition points. This implies that to adapt from a free-living directed state to one where the host imposes control over the relative value of C : N is plausible and in this aspect does not require much adjustment.

## 4. Discussion

Here we took the novel approach of applying FBA to model an evolutionary transition to endosymbiosis. Following an investigation into the free-living state of the cyanobacteria symbiont, predictions were made that charted the transition to endosymbiosis. The work focused on the metabolic changes that would occur, particularly in terms of the exchange reaction at the heart of the symbiosis. It accounted for both the symbiont whose metabolism was modelled directly but also considered the interest of the host through the ‘price’ of the compounds and compensation costs. In doing so, FBA modelling has been used to predict the metabolic transitions that may have occurred in an ancient, or more contemporary, evolutionary event.

Of interest are the possible evolutionary trajectories of the host and symbiont once a basic endosymbiotic relationship is established. Presumably, there are many possible alternatives to the formation of an obligate endosymbiosis, including loss of symbiosis, and these are determined by the changes that must occur in both partners. In this work, we have focused exclusively on the metabolic changes, and this predicts discontinuous changes in the exchange metabolites as the holobiont strives for higher growth rates and the control of the relationship changes. However, we emphasize that the modelling approach we have used does not include regulation, nor additional costs and benefits such as membrane production and maintenance or photo-protection, respectively.

There is one independent endosymbiotic event, which, like the primary endosymbiotic event, involved the uptake of cyanobacteria as the symbiont. The amoeba *Paulinella chromatophora* has been found to have an organelle-like structure, a chromatophore, which is derived from a cyanobacterium—*Synechococcus* [40]. There have been several, possibly 32, genes transferred to the nucleus [41], and these genes are biased towards a role in photosynthesis, for instance psaE which is a peripheral protein in photosystem I. This suggests that these transfers are examples of fully functional endosymbiotic gene transfers and because some of the proteins encoded are localized to the chromatophore, a protein import mechanism is implicated. There are several suggested mechanisms of protein import but its exact nature is currently unknown [42]. Relative to the primary endosymbiotic event this is a ‘recent’ event, but it is still ancient, occurring approximately 60 million years ago [43]. It highlights the propensity for cyanobacteria and protists to form endosymbioses that can in rare instances evolve to become an organelle.

Our predicted optimal exchange metabolites for the symbiosis are known to be exchanged in some natural endosymbioses. For instance, glutamate, along with glutamine and aspartate, is provided by the aphid to its bacterial endosymbiont [23,44]. Arginine metabolism, however, is often associated with symbioses without it being the actual exchange metabolite; for instance in the arbuscular mycorrhizal symbiosis arginine is converted to ammonium in the terminal arbuscule before being unloaded into the interfacial apoplast [45]. In addition to this example, ammonium is the nitrogen exchange metabolite in several other symbioses, including *Gunnera*–*Nostoc* [46], salamander eggs and green algae [47] and Rhizobia and legumes [48]. Ammonium, unlike arginine, does not contain any organic carbon; it could therefore be that the model's carbon compensation mechanism is only partially able to account for this cost to the host. Because the model only indirectly models the host, this is perhaps unsurprising. Ammonium was predicted as being the second-best metabolite after arginine, but evidence from natural endosymbioses implies that when the host is fully considered, this balance changes and ammonium is preferred. This assumes, however, that the exchange will be similar to current symbioses and it may be the case that a *Synechocystis*–*Paramecium* endosymbiosis would have an unusual exchange reaction.

Pyruvate as an exchange metabolite is unusual. There are a few examples where it is exchanged; for instance it is excreted by the bacterial symbiont of a luminescent fish [49]. The vast majority of symbioses, however, use simple carbohydrate sources instead; for instance, glucose and glycerol are exchanged between dinoflagellates and cnidarians [50,51], maltose between *Paramecium*–*Chlorella* [17], and malate between Rhizobia–legumes [48]. This may be because pyruvate has a central role in metabolism and therefore feedback regulation, which means that changing its concentration could have knock-on detrimental effects [52,53]. The model cannot consider this potential regulation constraint for pyruvate, because FBA modelling does not include regulation, which can lead to biologically implausible scenarios. Possible regulation conflicts affect many of the intermediates of glycolysis and the tricarboxylic acid cycle. For example, 3-phosphoglycerate has a positive feedback effect on photosystem protein synthesis; therefore, excess depletion could decrease photosynthesis [54]. The consequences of the complicated regulation systems of the major metabolic pathways need to be taken into consideration.

This work has predicted the optimum metabolic compound without the constraints of regulation. This is the first step required in understanding the coevolution process as it reveals what the symbiont, in particular, would be ‘aiming’ for. Any diversions from the metabolically optimum exchange would reveal additional restraints, either from biochemical regulation or conflict between the organisms, and indicates which partner is forced to bear the cost of the endosymbiosis. For instance, if pyruvate is not often exchanged in modern symbioses, then it indicates that the carbon-providing organism is forced to invest in further converting the carbon source, potentially because of host-controlled carbon transporters that could pull out pyruvate from the essential carbon reserves.

In addition, the model predicts a change from glutamate to arginine as the endosymbiosis progresses, and because arginine has the most markedly different metabolism, this reveals that perhaps the metabolic adaptation to mutualism is more extreme than to the initial symbiosis. This more severe change would, however, have the advantage of being a transition that could develop over time, whereas the initial symbiotic event is abrupt. The results of varying the C : N ratio and the degree of carbon compensation also support the idea that the initial adaptation is plausible and does not require much adjustment.

This work has demonstrated how FBA modelling can be applied to evolutionary questions. Parameter values are used that allow the metabolism of *Synechocystis* to be studied over a spectrum of cooperation. This analysis is analogous to the potential changes that the symbiont may undergo as it adapts from a free-living organism to living within a host. These predications are applicable to the primary endosymbiotic event and provide a mechanism by which metabolism of an ancient event can be inferred.

## Supplementary Material

File 1: Output file for standard conditions

## Supplementary Material

File 2: Output file for symbiotic state (exchanging pyruvate for guanine as an example)

## Data accessibility

The model, in both its free-living and symbiotic state, is included as supplementary data. FBA models are provided in a format conforming to the BiGG database format and are provided with example flux distributions to enable reproducibility.
